# Baseline demographic, clinical and immunological profiles of HIV-infected children at the Yaounde Gynaeco-Obstetric and Pediatric hospital, Cameroon

**DOI:** 10.11604/pamj.2014.17.87.3264

**Published:** 2014-02-03

**Authors:** Florence Soh Fru, Andreas Chiabi, Séraphin Nguefack, Evelyn Mah, Virginie Takou, Jean Baptiste Bogne, Marie Lando, Pierre-Fernand Tchokoteu, Elie Mbonda

**Affiliations:** 1 Yaounde Gynaeco-Obstetric and Pediatric Hospital, Cameroon; 2 Yaounde Gynaeco-Obstetric and Pediatric Hospital/Faculty of Medicine and Biomedical Sciences, University of Yaounde I, Cameroon

**Keywords:** Demographic, clinical presentation, immunological profile, HIV, children, Cameroon

## Abstract

**Introduction:**

Approximately 2.5 million children below 15 years are infected with the HIV virus, with 90% in sub-Saharan Africa. The Yaounde Gynaeco-obstetric and Pediatric hospital has been a treatment center for HIV since 2006. The aim of this study was to analyze the baseline demographic, clinical and immunologic characteristics of the children with the HIV infection in this hospital.

**Methods:**

It was a retrospective, cross- sectional and analytic study, carried out between January and April 2011 which included 61 HIV positive children aged 0-15 years. The socio-demographic, clinical and immunologic data were obtained from their medical records.

**Results:**

Most (52.5%) of the children studied were above 60 months of age with a mean age of 71 months. Most (57.4%) were females. Mother-to-child transmission was the principal mode of contamination in 88.5% of cases. More than half of their mothers (55.7%) did not receive antiretroviral prophylaxis during pregnancy and labor. Common clinical findings included prolonged fever (44.6%), malnutrition (37.6%), lymphadenopathy (34.4%), respiratory tract infections (34.4%) and diarrhea (24.5%). Diagnosis was confirmed by HIV serology for most of the patients (93.4%). Polymerase chain reaction served as method of diagnosis in only 6.6% of the cases. HIV 1 was the predominant viral type. More than half of the children (52.5%) were seen at an advanced stage of the disease.

**Conclusion:**

HIV screening during pregnancy and prevention of mother-to-child transmission should be reinforced in this context, and fathers of HIV-infected children should be encouraged to go for HIV testing.

## Introduction

According to UNAIDS report of 2012, it was estimated that 34 million people in the world live with the HIV virus, two thirds of whom are found in sub Saharan Africa [1]. The number of AIDS related deaths was estimated at 1.7 million, 1.2 million of which occurred in sub Saharan Africa. Approximately 4% of all deaths of children younger than 5 years are attributable to HIV/AIDS [2]. The clinical expression of HIV infection in children is highly variable and non-specific. Some HIV-positive children develop severe HIV-related signs and symptoms in the first year of life. Others may remain asymptomatic or mildly symptomatic and may survive for several years [3]. Clinical signs permit suspicion of HIV infection but specific biological tests are needed to make the definitive diagnosis.

In a study in India on 109 HIV- Infected children (82 boys, 27 girls), the median age at presentation was 48 months (range: 0.75 months-180 months). Eighty one (74.3%) children acquired the infection vertically, and 91 (83.5%) children were symptomatic at presentation. The common symptoms in the former were failure to thrive (81.3%), recurrent fever (73.6%), diarrhea (50.5%) and recurrent or persistent pneumonia (44%), and all children had a poor nutritional status at baseline [4].

In a cohort study of the socio-demographic characteristics of a group of HIV-positive Nigerian children, most of the parents were in the 21- 40 age group, and more than half of the parents had secondary education. As concerns the HIV status, 34.5% of the fathers were HIV-negative, 25.4% HIV-positive and in 40% the status was not known; 70.9% of the mothers were HIV-positive and 21.1% HIV-negative [5]. The Yaounde Gynaeco-Obstetric and Pediatric Hospital (YGOPH) has been a treatment center for HIV infection since 2006. We decided to carry out this baseline study whose objective was to analyze the baseline socio-demographic, clinical and immunologic characteristics of the HIV-infected children followed up. The results of this study will help improve the management of these children in this referral hospital.

## Methods

This was a retrospective cross-sectional analytic study, from January to April 2011. We enrolled 61 HIV-positive children aged 0-15 years followed up in the pediatric unit of the YGOPH between 2006 and 2010.

Their socio-demographic, clinical and immunologic data were obtained from their medical files and recorded on questionnaires. The data collected included age, sex, presenting symptoms, anthropometric measurements, results of the physical examination and laboratory investigations. Information concerning the HIV status, age and profession of their parents was noted. The past medical history of the patients including perinatal history, mode of feeding in infancy, past history of transfusion or scarifications, clinical and immunological stages was also noted. The patients were classified clinically using the WHO 2006 classification [3]. The diagnosis of HIV infection in some of our patients less than 18 months was done by the detection of proviral HIV-DNA by PCR. The mononuclear cells from which the proviral DNA was extracted was obtained from dry blood spots collected at the blood bank of YGOPH. The tests were done in the laboratory of the Chantal Biya International Research Center (CIRCB) which is a reference laboratory. However most of the patients were diagnosed by serology. The serologies were done using Determine which is a rapid test (Alere medical co.Ltd Japan). It is an immunochromatic test which gives either a positive or negative result. Secondly the Immunocomb test (Orgenic Israel) which is an immunoenzymatic test, was done to determine the viral type (HIV1or 2)

The CD4 counts were done in the hospital laboratory, using BD Facscount flow cytometer (England). The patients were classified into 3 groups; those with no immunologic deficit (CD4% ≥ 25%), moderate immunologic deficit (CD4% = 15-24%) and severe immunologic deficit (CD4%< 15%) [3].

Some of the patients whose files had incomplete information and whose telephone numbers were recorded, were contacted by phone to obtain the missing information. Data analysis was done using the statistical package for social sciences version 16.0.

## Results

We enrolled 61 patients who met up with our inclusion criteria. There were 26 males (42.6%) and 35 females (57.4%) giving a sex ratio of 0.74.

**Entry point at diagnosis:** Most of the patients 41(67.2%) were diagnosed at the outpatient consultation based on their clinical presentation or following referral from other health institutions, 14(22.9%) during hospitalization, 4(6.6%) from prevention of mother to child transmission (PMTCT) services and 2 (3.3%) after screening following the diagnosis of a case in the family.

**Age of the patients:** Most of the patients were more than 60 months of age, with a mean age was 71 months. There was a significant difference in the age distribution (P = 0.000) (Table 1).

**Table 1:** Distribution of patients by age

Age groups	Number	Percentage (%)
< 18 months	4	6.6
18-60 months	25	41.0
> 60 months	32	52.5
**Total**	61	100.0

**Mode of contamination:** Mother to child transmission (MTCT) was the principal mode of contamination in 54(88.5%) and unknown in 7(11.5%). patient.

**PM TCT during pregnancy:** Concerning PMTCT during pregnancy, 34 (55.7%) of the mothers did not receive antiretroviral (ARV) prophylaxis during pregnancy and labor, and in 23 (37.7%) files it was not indicated whether ARVs were taken or not.

**Mode of diagnosis:** The diagnosis was made by HIV serology for most of the patients, 56(91.8%). PCR served as method of diagnosis in only 8.2% of the cases, and both methods were used in 1 case (1.6%). Most of the patients, 52(85.2%) were infected with the HIV1, and 9(14.8%) had both the HIV1&2.

**HIV status of the parents:** Most of the fathers did not know their HIV status and 6.6% were confirmed discordant couples (Table 2).

**Table 2:** Distribution of patients according to HIV status of the parents

	Positive (number/%)	Negative (number/%)	Unknown (number/%)	Total (number/%)
Father	9 (14.8%)	4(6.6%)	22(36.1%)	35(57.4%)
Mother	0(0.0%)	2(3.3%)	24(39.3%)	26(42.6%)
**Total**	9(14.8%)	6(9.8%)	46(75.4%)	61(100.0%)

**Age distribution of the parents:** Most of the mothers were in the 20 -34 age group and the fathers in the greater than 34 years age group. There was a statistically significant difference in the age group distribution of both the mothers (P = 0.001) and the fathers (P-value= 0.016) (Table 3).

**Table 3:** Age distribution of the parents

Parents Age(years)	Mother (N = 61)		Father (N = 61)	
	Number	Percentage(%)	Number	Percentage(%)
**< 20**	0	0.0	0	0.0
**20-34**	34	55.7	10	16.4
**>34**	11	18.0	24	39.3
**Unknown**	16	26.3	27	44.3
**Total**	61	100.0	34	100.0

**Profession of parents**: Most of the mothers whose profession could be known were unemployed (29.5%) while most of the fathers whose profession could be known were self-employed (31.1%) (Table 4).

**Table 4:** Profession of parents

Profession of parents	Mothers (N = 61)		Fathers (N = 61)	
	Number	Percentage(%)	Number	Percentage(%)
Civil servants	8	13.1	9	14.8
Self-employed*	9	14.8	19	31.1
Unemployed	18	29.5	2	3.3
Student	3	4.9	0	0.0
Unknown	23	37.7	31	50.8
**Total**	61	100.0	61	100.0

* Parents who had their own private businesses

**Common clinical findings**: The most common clinical findings were fever, malnutrition, lymphadenopathy, and respiratory infections (Table 5).

**Table 5:** Common clinical findings

Clinical Manifestations	Number	Percentage(%)
Fever	27	44.6
Malnutrition	23	37.6
Lymphadenopathy	21	34.4
Respiratory tract infection	21	34.4
Diarrhea	15	24.5
Ear infection	12	19.6
Parotiditis	10	16.4
Splenomegaly	6	9.8
Zona	5	8.2
Thrush	4	6.6
Anemia	3	4.9
Hepatomegaly	2	3.3
Vomiting	2	3.3
Others**	6	9.8

*A patient could have had more than one symptom

** Abcess, irritability, nodal tuberculosis, pharyngitis, psychomotor regression and varicella

**Clinical staging:** According to the WHO 2010 clinical classification, most of the patients were at clinical stage 2 and stage 3 (Table 6).

**Table 6:** Classification of HIV-associated clinical disease

Clinical taging	Number	Percentage (%)
1	8	13.1
2	22	36.1
3	23	37.7
4	8	13.1
**Total**	**61**	**100.0**

**Immunological staging:** More than half of the children were seen at an advanced stage of the disease with severe immunologic deficit (Table 7).

**Table 7:** Immunological stage

Immunological stage	Number	Percentage(%)
No deficit ≥ 25%	11	18.0
Moderate deficit 15-24%	18	29.5
Severe deficit < 15%	32	52.5
**Total**	61	100.0

## Discussion

The age range of the patients was 12 to 180 months, with most of them more than 60 months old. Most of them were therefore diagnosed late. Late diagnosis was found in India where the mean ages were 54.0±34.8 months [6], and 54 months [7], in Senegal the median age was 60 months [8] and in Rwanda the median was 56 months [9]. In contrast, a review of pediatric HIV among hospitalized children in a Nigerian Teaching Hospital found a lower mean age at diagnosis of 17±23.2 months [10]. It is worth noting that even the 4 children whose diagnosis was made by PCR were 12months or older at the time of diagnosis.

The sex ratio of the patients was 1:1.35. A similar sex ratio of 1:1.4 were found in Nigeria [11] Other studies noted a male sex predominance with sex ratios of I.3:1, 1.1:1, 3: 1, 3:1 and 6:1 [4, 6, 12–14].

Mother—to—child transmission was the principal mode of contamination in 88.5% of the patients. This observation is similar to what was found in India and Nigeria [7, 10, 15]. We observed that PMTCT was not done in 55.7% of cases. This figure is probably under evaluated since in a good number of patients (37.7%) we could not say whether prophylaxis was given or not. Considering the fact that this is the main strategy for the prevention of pediatric HIV infection, this observation calls for more active interventions in this domain. However this might not reflect the present situation as most of the children were above 5 years whereas better strategies to reduce MTCT have been put in place only within the past 5 years.

The diagnosis was made by HIV serology in most of the patients, in 91.8%. PCR served as method of diagnosis in only 8.2% of the cases.

The HIV type of all the patients was known and most of them had the type 1 virus (85.2%). In a previous study in Cameroon all the patients had only HIV type 1 [16]. In Nigeria similar results were found with the majority (93.9%) of their patients having HIV1 [11]. These findings are not surprising since HIV 1 is the more rapidly replicating type with a higher risk of transmission. The rest of the patients had a combination of type 1and 2 viruses and none had only type 2 virus. Although 2 strains of HIV have been identified, most patients who have AIDS are positive for HIV type 1. HIV 2 infection is most commonly observed in West Africa and mother-to-child transmission of HIV2 is rare thus responsible for very little or no cases of pediatric infection [17]. The viral group was not known in any of the cases. This is because the laboratory where the testing was done did not do grouping. All the parents were within the child bearing age with most mothers in the 20 -34 age group and fathers in the greater than 34 years age group. In Nigeria, similar results were found with mothers aged 15 - 43years and the fathers 27-55years [18]. Sexual transmission being the principal mode of transmission of HIV, it is logical that the parents from whom the children principally got the infection were within the sexually active age group. Most of the mothers (29.5%) whose profession could be known were unemployed while most of the fathers (31.1%) whose profession could be known were self-employed. In Senegal in 2003, 30% of the fathers were middle class workers and the rest unemployed [8].

The HIV status of more than half of the mothers was known and all of them were positive, whereas the HIV status of most of the fathers was not known. None of the mothers was known to be HIV-negative. In Senegal, 99% of the mothers and 15.3% fathers were positive [8]. Four (6.6%) of the children with HIV-negative fathers had HIV-positive mothers.

In Nigeria in 2008 94.5% of the mothers were sero-positive and 5.5% sero-negative. There were 15 discordant couples with all the mothers positive, while the all fathers were negative [18].

Most of the patients (93.4%) were symptomatic. Other studies found HIV- infected children to be symptomatic in 83.5% [4], 65.6% [7] and 42% [6]. The most common clinical manifestations in our patients were prolonged fever (44.6%), malnutrition (37.6%) lymphadenopathies (34.4%) and respiratory infections (34.4%). The common clinical finding in a study carried out in 4 hospitals in Yaoundé in 2002 were anemia (85%), prolonged fever (63%), diarrhea >1 month (46%), weight loss or cachexia (43%) [16]. In India, the main clinical manifestations were pulmonary tuberculosis (55%), oral candidiasis (43%), and recurrent respiratory tract infection (26%) and skin infections (21%) [19]. In Dar es Salaam, weight loss, generalized lymphadenopathy, recurrent fever and prolonged diarrhea were the main clinical predictors of HIV infection among preschool children [20], and in Ibadan weight loss (62.5%), prolonged fever (55.4%), generalized lymphadenopathy (48.6%) and chronic cough (45.4%) were found to be the most common clinical features [10]. We noted that malnutrition, fever and respiratory tract infections were very frequent in almost all of the studies but neurologic signs and zona were rare. According to the WHO 2010 immunological classification, the majority of our patients were at stages 2 and 3 disease. These two stages correspond to symptomatic disease which is quite understandable since most children were brought to the hospital only when they had signs and symptoms of disease. In Ibadan using this same classification, 70.6% of their patients were at clinical stage 3 and 4 [10], whereas in India 45.1% of the patients were in WHO clinical stages 3 and 4 at the time of presentation [14]. These findings indicate that most children were also seen at an advanced stage of the disease.

The immunological classification was done following the CDC 1994 immunologic categories [3], which accompanied the results of the test done in the hospital laboratory. More than half of them had severe immune deficiency. Using a similar classification in Nigeria 44.5% of the study population had severe immunosuppression [10]. This does not reflect the exact level of immuno-depression since this classification had been updated in 2006. It can however reflect the advanced stage of disease at the time of management. This can be explained by the fact that before onset of clinical manifestations there is first of all viral multiplication resulting in the destruction of CD4 cells.

The limitations of this study are, the retrospective study design in which many files had incomplete information resulting in a small sample size. Some patients were previously on ARV treatment which might have had some influence on the clinical manifestations at the time of presentation in our hospital. Also, we noticed that the viral groups were not determined probably due to technical difficulties.

## Conclusion

Mother to child transmission is the most common mode of contamination of HIV infection in children presenting at our hospital. Presentation in hospital is late when the children are already symptomatic, and at late clinical and immunologic disease stages. These findings call for reinforcement of PMTCT at all levels of the health system and greater sensitization of the general public on the prevention of HIV infection.
